# Swinhoeic acid from *Potentilla fragarioides* ameliorates high glucose-induced oxidative stress and accumulation of ECM in mesangial cells via Keap1-dependent activation of Nrf2

**DOI:** 10.1080/13510002.2022.2134755

**Published:** 2022-10-19

**Authors:** Huankai Yao, Min Kong, Dan Du, Fengwei Ai, Jindong Li, Yan Li

**Affiliations:** a Department of Microbial and Biochemical Pharmacy, School of Pharmacy & Jiangsu Key Laboratory of New Drug Research and Clinical Pharmacy, Xuzhou Medical University, Xuzhou, People’s Republic of China; b Department of Pharmacy, Taizhou People’s Hospital, Taizhou, People’s Republic of China

**Keywords:** Swinhoeic acid, diabetic nephropathy, mesangial cells, high glucose, self-limited proliferation, oxidative stress, extracellular matrix, Nrf2

## Abstract

**Objectives:**

Diabetic nephropathy (DN) is one of the most common microvascular complications of diabetes mellitus. Oxidative stress resulting from high glucose promotes accumulation of ECM and development of DN. Activation of Nrf2 could attenuate oxidative stress and following accumulation of ECM. To find novel therapy for DN, we explored the effects of swinhoeic acid from *Potentilla fragarioides* on mesangial cells under high glucose and underlying mechanisms.

**Methods:**

CCK-8 and BrdU incorporation assays for survival of mesangial cells gave the concentration of swinhoeic acid in following investigations. ROS, MDA, SOD and CAT were determined. And ECM proteins and their upstream regulators TGF-β_1_ and CTGF were detected using ELISA assays. Activation of Nrf2 was explored by immunofluorescence staining together with luciferase reporter assay. To demonstrate the role of Nrf2 activation, siRNA interference was performed. And co-immunoprecipitation assay was used to elucidate swinhoeic acid affects the interaction between Keap1 and Nrf2.

**Results:**

Swinhoeic acid at 10 and 20 μM attenuated oxidative stress and accumulation of ECM in mesangial cells under high glucose. Itactivated Nrf2 in a Keap1-dependent manner, which was involved in its effects.

**Conclusion:**

Swinhoeic acid ameliorates oxidative stress and accumulation of ECM resulting from high glucose in mesangial cells via activating Nrf2 in Keap1-dependent manner.

## Introduction

Diabetic nephropathy (DN) is one of the fatal microvascular complications of diabetes mellitus and has become the main cause of end-stage renal disease (ESRD) worldwide [1]. In clinic, it is characterized as proteinuria and progressive renal function impairment [2]. Histopathological detection has revealed the kidney of DN patients displayed basement membrane thickening and mesangial expansion due to the accumulation of extracellular matrix (ECM) and self-limited proliferation of glomerular mesangial cells [3]. Pathogenesis of DN indicates high glucose stimulates the proliferation of mesangial cells at the early stage followed by hypertrophy since the proliferated cells are arrested [4,5]. And the aberrant metabolism of glucose has led to the generation of excessive reactive oxygen species (ROS), which initiates the excessive synthesis of transforming growth factor β_1_ (TGF-β_1_) in addition to oxidative stress [6]. As the key fibrogenic cytokine, TGF-β_1_ enhances the accumulation of ECM via increasing its secretion and stability as well as suppressing its degradation [7]. Without timely prevention, glomerulosclerosis and renal fibrosis will take place followed by proteinuria and renal function impairment, which will finally develop into ERSD and result in the employment of dialysis and renal transplantation to reduce renal failure [8,9].

Nuclear factor erythroid 2-related factor 2 (Nrf2) is a transcription factor that affords the transcriptional regulation of target genes including antioxidant enzymes [10]. Under basal condition, Nrf2 is captured by Kelch-like ECH-associated protein-1 (Keap1) in cytosol through the formation of Keap1-Nrf2 complex, which can promote the ubiquitination and proteasomal degradation of Nrf2 [11]. However, when exposed to oxidants or electrophiles, the cysteine residues in Keap1 will be affected and the conformation of keap1 will change, which results in the dissociation of Keap1-Nrf2 complex [12]. Then the free Nrf2 in cytosol will translocate into nucleus and promote the transcription via binding to the antioxidant response elements (ARE) sequence in the promoter region of target genes [13]. In the progression of DN, high blood glucose has caused oxidative stress and fibrosis by up-regulating TGF-β_1_ and ECM expression in mesangial cells [14]. Therefore, activating Nrf2 shows a potential strategy to attenuate DN via inhibiting oxidative stress followed by fibrosis.

**Figure 1.**
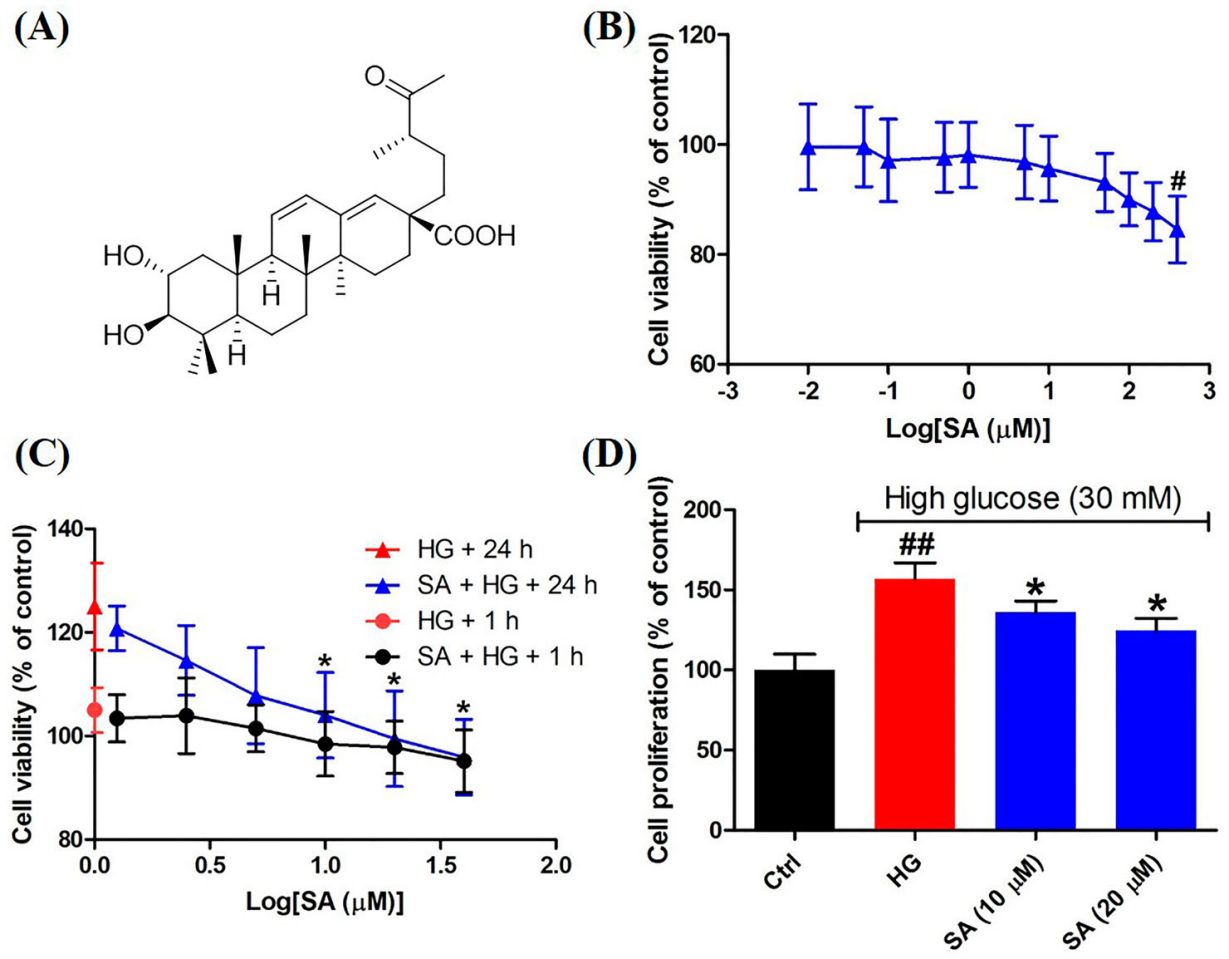
Effects of swinhoeic acid on the survival of mesangial cells with or without high glucose. (A) Chemical structure of swinhoeic acid. (B) Effects of swinhoeic acid on the viability of mesangial cells cultured in normal medium. (C) Effects of swinhoeic acid on the viability of mesangial cells under high glucose after 1 or 24 h. (D) Effects of swinhoeic acid on the proliferation of mesangial cells induced by high glucose. n = 3, ^#^*P* < 0.05 and ^##^*P* < 0.01 *vs* control group, *P < 0.05 *vs* high glucose group.

In the discovery of Nrf2 activators, phytochemicals play a crucial role such as sulforaphane, curcumin, resveratrol and so on [15]. *Potentilla fragarioides* is a perennial herb grown in most areas of China. The whole plant is used to attenuate toothache and arthritis in Chinese folklore [16]. However, phytochemicals in this plant are not quite clear. In our interest to find Nrf2 activators from nature, we have identified major phytochemicals [16] and evaluated their effects on Nrf2 activation with the potential therapy for DN. Of these compounds, swinhoeic acid (Figure 1(A)) as a rare triterpenoid came into sight. Though this compound was found in *Rubus swinhoei* for the first time [17], to the best of our knowledge, there is no investigation on its bioactivity. Herein we reported the effects of swinhoeic acid on human glomerular mesangial cells under high glucose and relevant mechanisms.

## Materials and methods

### Chemicals and reagents

Swinhoeic acid (SA) was prepared from *Potentilla fragarioides* with the purity of more than 98% analyzed by HPLC as our previous description [16]. Cell counting kit-8 (CCK-8) assay kit was supplied by Dojindo Laboratories (Kumamoto, Japan). Bromodeoxyuridine (BrdU) cell proliferation assay kit was obtained from Shanghai Boyun Biotech (Shanghai, China). DyLight 594-conjugated secondary antibody was a product of EarthOx Life Sciences (Millbrae, CA). ROS assay kit, malondialdehyde (MDA) assay kit, superoxide dismutase (SOD) assay kit, catalase (CAT) assay kit, 4′,6-diamidino-2-phenylindole (DAPI) staining solution, rabbit IgG, nuclear and cytosolic proteins extraction kit, bicinchoninic acid (BCA) protein assay kit, and enzyme-link chemiluminescence (ECL) assay kit were purchased from Beyotime Biotechnology Institute (Shanghai, China). N-acetyl-cysteine (NAC) and aminotriazole (ATZ) were provided by Merck (Darmstadt, Germany). Human haemoxygenase-1 (HO-1), collagen IV, fibronectin, laminin, TGF-β_1_ and connective tissue growth factor (CTGF) ELISA kits were obtained from Colorfulgene Biotechnology (Wuhan, China). The primary antibodies including Nrf2, Keap1, β-actin and lamin B1 together with horseradish peroxidase-conjugated secondary antibody were products of Abcam (Cambridge, UK). The ARE-luciferase reporter plasmid (pGL4.37[luc2P/ARE/Hygro]), renilla luciferase reporter plasmid (pRL-TK) and dual-luciferase reporter assay system were supplied by Promega (Madison, WI). Lipofectamine 2000 and the Nrf2 activator, sulforaphane (SFN), were purchased from Thermo Fisher Scientific (Waltham, MA). Negative control siRNA (NC-siRNA) and Nrf2-siRNA were obtained from Santa Cruz Biotechnology (Dallas, TX). Protein A agarose was furnished by Santa Cruz Biotechnology (Santa Cruz, CA).

### Cell culture and treatment

Human glomerular mesangial cells were supplied by American Type Culture Collection (ATCC, Manassas, VA) and maintained in DMEM including 10% FBS, 100 U/mL penicillin and 100 mg/mL streptomycin at 37°C under the condition of 5% CO_2_ and 95% air. Then the cells were grouped as control group (Ctrl), high glucose group (HG), and experimental groups, and starved in the presence of serum-free medium for 24 h. After that, cells experimental groups were incubated with certain swinhoeic acid or sulforaphane in the medium including high glucose (30 mM) for 1 or 24 h. But cells in high glucose group were just treated with high glucose. And those in control group were maintained in DMEM with normal glucose (5.6 mM).

### CCK-8 assay

CCK-8 assay was implemented to reveal the effects of swinhoeic acid on the survival of mesangial cells with or without high glucose. In brief, the cells were seeded in 96-well microplates and treated as above. Then incubation with 10 μL CCK-8 solution was performed at 37°C for 1 h. The absorbance was recorded on a microplate reader (Bio-Rad, Hercules, CA) at 450 nm.

### Brdu incorporation assay

To disclose the effects of swinhoeic acid on the self-limited proliferation of mesangial cells, BrdU incorporation assay was performed using the assay kit. According to the supplier’s instruction, the mesangial cells in logarithmic growth phase were seeded in 96-well microplates at the density of 1 × 10^5^/mL. After cofluence about 80%, the medium was removed and the cells were washed with PBS for three times. After synchronized for 4 h, the cells were treated as above and incubated with 10 μL BrdU solution (10 μM) at 37°C for 3 h, and then with FixDenat at room temperature for 30 min after clearing the solution. Following the addition of anti-BrdU-POD solution, the incubation was carried out at room temperature for 90 min. After exposed to substrate solution, the absorbance was read on the microplate reader at 370 nm.

### Production of intracellular ROS

The production of intracellular ROS in mesangial cells was measured using the assay kit. Briefly, the treated cells were exposed to 2′,7′-dicholorofluorescein diacetate (DCFH-DA) at 37°C in the dark for 20 min. After rinsed with serum-free medium, the fluorescence intensity was detected on a fluorescence microplate reader (Molecular Devices, San Jose, CA) at the excitation wavelength of 488 nm and emission wavelength of 525 nm.

### MDA content

The content of MDA in glomerular mesangial cells was detected using the MDA assay kit. The treated cells were lysed on ice using RIPA buffer. After centrifugation, the supernatant was collected and treated according to the supplier’s instruction. Then the absorbance was determined on a micropale reader at 523 nm.

### SOD and CAT activity

Colorimetric method was used to measure the activity of SOD and CAT in mesangial cells. After lysis on ice, the treated cells were centrifuged and the supernatant was collected for further analysis using the assay kits. According to the supplier’s protocols, the samples were handled and the absorbance was monitored at 560 nm for SOD activity as well as 520 nm for CAT activity. The relative enzyme activity was calculated from the absorbance of experimental groups versus that of control group.

### ELISA assay

ELISA kits were employed to reveal the synthesis of HO-1 and major ECM proteins such as collagen IV, fibronectin and laminin as well as the secretion of cytokines including TGF-β_1_ and CTGF. After being treated as above, the cells were centrifuged and the supernatant was sucked as samples. Then the samples were treated in the light of the supplier’s instructions, and the absorbance was recorded at 450 nm. The levels of HO-1, collagen IV, fibronectin, laminin, TGF-β_1_ and CTGF were obtained from the absorbance against that of control group.

### Immunofluorescence staining

To locate Nrf2 in mesangial cells, immunofluorescence assay was implemented. Briefly, the cells were cultured in 12-well microplates and there was one coverslip in each well. After treatment as above, the cells were fixed with 4% paraformaldehyde at 4 °C for 15 min and permeated using PBS containing 0.1% Triton X-100 for 10 min. After being blocked with 1% BSA for 30 min, the cells were incubated with primary antibody against Nrf2 (1:200, v/v) at 4 °C overnight, and then exposed to DyLight 594-conjugated secondary antibody (1:200, v/v) at 37°C in the dark for 1 h after washed with PBS. The cells were stained with DAPI solution in the dark. The coverslips were mounted on glass slides and the image was captured under a fluorescence microscope (Olympus, Tokyo, Japan).

### Dual luciferase reporter assay

To evaluate the transcription capacity of Nrf2, dual luciferase reporter assay was employed herein. The mesangial cells were co-transfected with ARE-luciferase reporter plasmid (pGL4.37[luc2P/ARE/Hygro]) and renilla luciferase reporter plasmid (pRL-TK) using lipofectamine 2000. Then the luciferase activity in the treated cells was detected using the dual luciferase reporter assay system on a Promega GloMax luminometer (Madison, WI). The capacity of Nrf2 to bind to ARE was assessed through the relative luciferase activity from normalizing the firefly luciferase activity versus the renilla luciferase activity.

### Total and nuclear proteins extraction

To extract the total proteins, the treated mesangial cells were lysed on ice using RIPA buffer. After centrifugation, the supernatant was collected and quantified using BCA protein assay kit. To obtain nuclear proteins, the treated glomerular mesangial cells were harvested and handled as our previous description [18]. At last the protein concentration of the supernatant was determined by BCA assay kit.

### Western blot analysis

The extracted total and nuclear proteins were resolved on 15% sodium dodecyl sulfate polyacrylamide gel electrophoresis (SDS-PAGE) and transferred to polyvinylidene fluoride (PVDF) membranes. After blockage with nonfat milk, the membranes were incubated with specific primary antibodies including Nrf2 (1:1000), β-actin (1:1000) and lamin B1 (1:10,000) at 4°C overnight. After rinsed with 0.1% Tween-20 buffer, the membranes were exposed to horseradish peroxidase-conjugated secondary antibody (1:2000) at room temperature. The bands were observed using ECL substrate and densitometric analysis was implemented using ImageJ software (NIH, Bethesda, MD).

### SiRNA interference assay

To elucidate the role of Nrf2 activation in the effects of swinhoeic acid, siRNA interference was implemented. The glomerular mesangial cells were transfected with NC-siRNA or Nrf2-siRNA in the light of the supplier’s protocol. And western blot analysis was used to indicate the successful transfection. Then the cells were treated as above, and ROS, MDA, SOD and CAT together with collagen IV, fibronectin, laminin, TGF-β_1_ and CTGF were detected respectively.

### Co-immunoprecipitation assay

To demonstrate the interaction between Keap1 and Nrf2 in presence of swinhoeic acid, co-immunoprecepitation assay was performed as our previous description [16]. In short, the treated mesangial cells were lysed on ice and centrifuged to obtain the supernatant. After preclear reaction using suspended protein A agarose, the samples were exposed anti-Nrf2 or rabbit IgG at 4°C for 1 h. Following the addition of protein A agarose and shaking overnight, centrifugation at 4°C was conducted to obtain the immunoprecipitates. After removing the supernatant carefully, the pellets were washed with lysis buffer and suspended in SDS loading buffer to be boiled for 5 min. Western blot analysis was used to examine the precipitated proteins.

### Statistical analysis

The experimental data were shown as mean ± standard deviation. GraphPad Prism 5.0 (San Diego, CA) was employed for statistical analysis. Shapiro–Wilk normality test was performed to validate the data followed normal distribution. Then one-way analysis of variance (one way ANOVA) followed by Tukey test was performed for multiple comparisons and student’s *t*-test were conducted for single comparisons. *P* < 0.05 was considered to be significant in statistics.

## Results

### Swinhoeic acid inhibits the self-limited proliferation of mesangial cells induced by high glucose

As shown in Figure 1(B), swinhoeic acid didn’t show significant cytotoxic effects on mesangial cells cultured in normal medium when its concentration is lower than 400 μM. However, compared to high glucose group, swinhoeic acid remarkably reduced the increasing cell viability from the concentration of 10 μΜ after 24 h though the viability was not obviously affected by swinhoeic acid at those concentrations after 1 h (Figure 1(C)). These results gave us the indication for the concentration of swinhoeic acid in following investigations. To further validate the inhibitory effects of swinhoeic acid on the self-limited proliferation of mesangial cells, BrdU incorporation assay was implemented. As a result, high glucose has induced the self-limited proliferation of mesangial cells, which was inhibited by swinhoeic acid at both 10 and 20 μΜ (Figure 1(D)).

### Swinhoeic acid attenuates oxidative stress resulting from high glucose in mesangial cells

**Figure 2.**
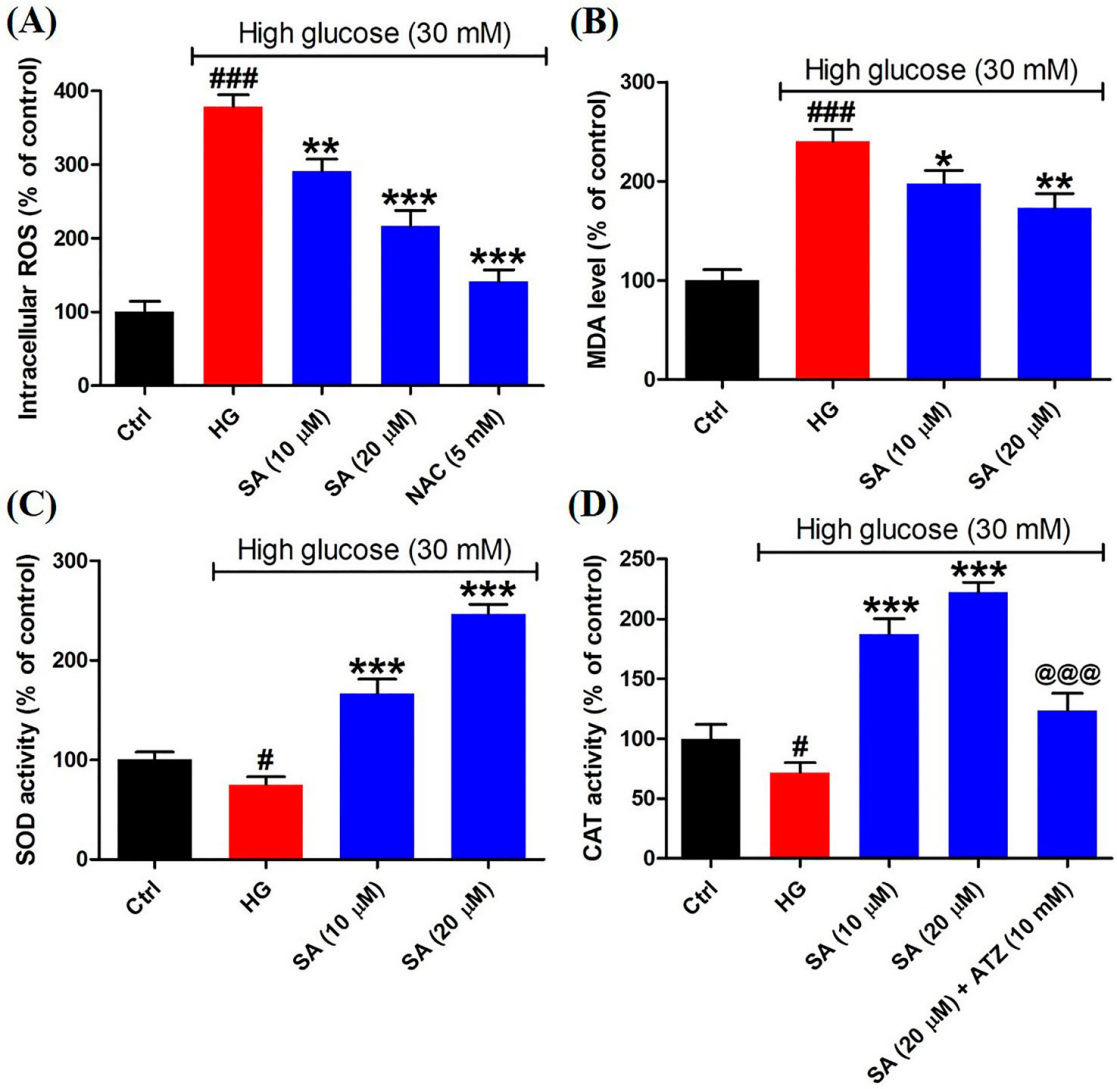
Effects of swinhoeic acid on the oxidative stress in mesangial cells under high glucose. (A) Intracellular ROS level. (B) MDA level. (C) SOD activity. (D) CAT activity. *n* = 3, ^#^*P* < 0.05 and ^###^*P* < 0.001 *vs* control group, *P < 0.05, **P < 0.01 and ***P < 0.001 *vs* high glucose group, ^@@@^*P* < 0.001 *vs* SA (20 μM) group.

To reveal the redox status in mesangial cells, the intracellular ROS was detected herein. The results indicated high glucose resulted in the overproduction of ROS (379.0 ± 15.8%) compared with control group (100.0 ± 14.5%), whereas the ROS level was decreased markedly after exposed to certain swinhoeic acid (291.6 ± 16.0% for 10 μM and 216.8 ± 21.0% for 20 μM). Meanwhile, in the presence of NAC, the ROS inhibitor, overproduction of ROS was reduced remarkably (141.5 ± 15.8%) (Figure 2(A)), which indicated ROS was generated in mitochondria and detected by the assay kit. As the product of lipid peroxidation, MDA content was monitored herein. The results showed high glucose led to the increment of MDA content sharply (240.1 ± 12.1%), while swinhoeic acid at 10 and 20 μM can significantly reduce the MDA content as 197.8 ± 13.1% and 173.0 ± 14.5%, respectively (Figure 2(B)). Meanwhile, swinhoeic acid could activate SOD (166.7 ± 14.3% and 246.4 ± 9.7%) and CAT (187.2 ± 12.9% and 222.2 ± 8.3%), which were inhibited under high glucose (75.2 ± 7.9% for SOD and 71.6 ± 8.3% for MDA) (Figure 2(C,D)). In addition, the elevated CAT activity by swinhoeic acid was obviously reduced when exposed to ATZ at 10 mM (123.4 ± 14.6%), which validated effectivity of the assay kit. These findings demonstrated swinhoeic acid could attenuate oxidative stress induced by high glucose in mesangial cells.

### Swinhoeic acid reduces the accumulation of ECM induced by high glucose in mesangial cells

**Figure 3.**
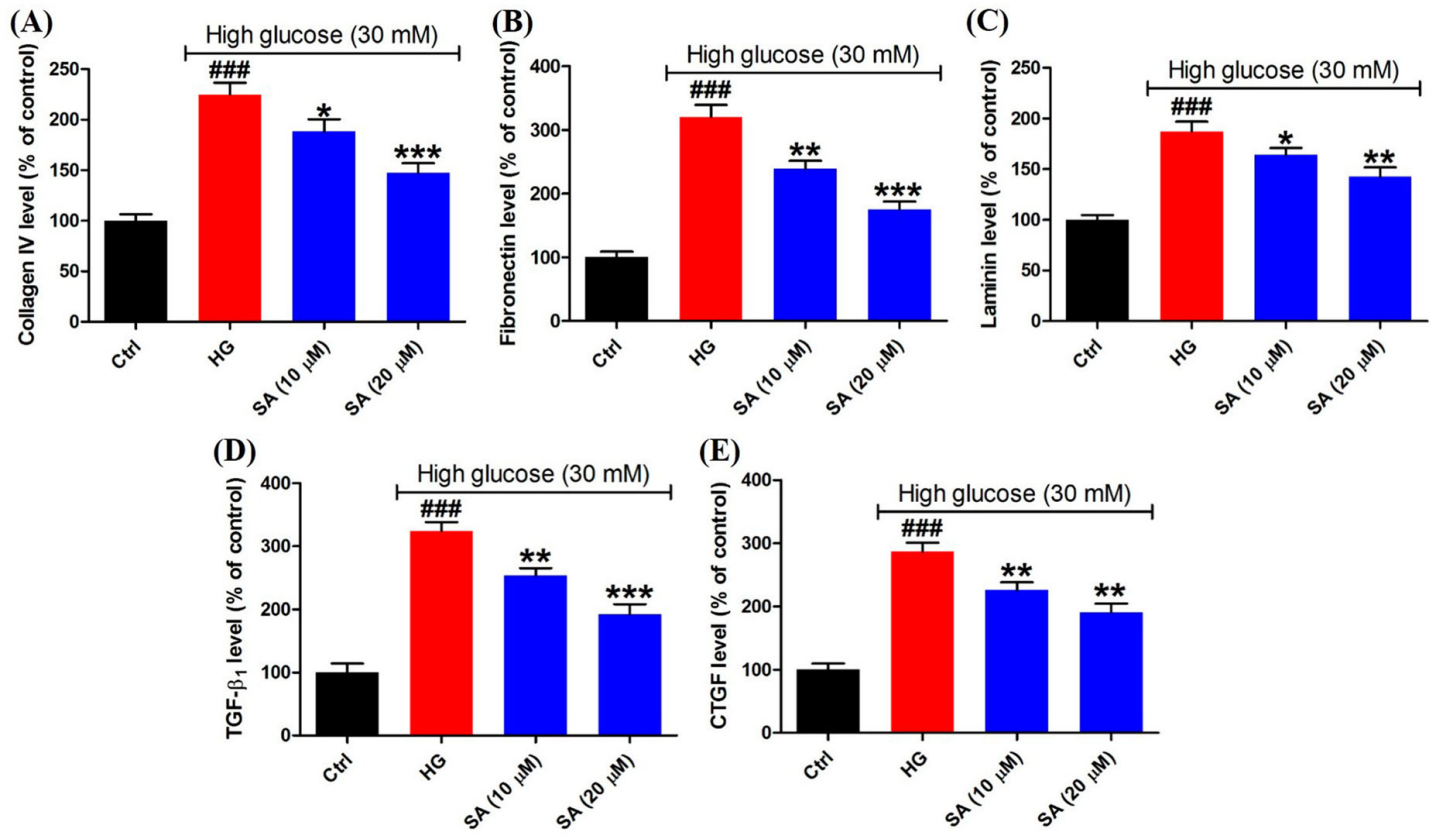
Effects of swinhoeic acid on the accumulation of ECM in mesangial cells under high glucose. (A)–(C) Synthesis of ECM proteins including collagen IV, fibronectin and laminin. (D)–(E) Secretion of TGF-β_1_ and CTGF. *n* = 3, ^###^*P* < 0.001 *vs* control group, *P < 0.05, **P < 0.01 and ***P < 0.001 *vs* high glucose group.

To evaluate the accumulation of ECM, the synthesis of ECM proteins including collagen IV, fibronectin and laminin was measured using the ELISA kits. As a result, high glucose has given rise to the excessive synthesis of collagen IV (224.5 ± 12.0%), fibronectin (320.3 ± 18.8%) and laminin (187.0 ± 9.8%). However, in the presence of 10 or 20 μM swinhoeic acid, the levels of collagen IV, fibronectin and laminin were decreased to 188.3 ± 12.1%, 238.6 ± 12.7% and 164.1 ± 6.8% or 147.3 ± 9.5%, 175.1 ± 12.2% and 142.7 ± 9.0% (Figure 3(A–C)). At the same time, after being exposed to high glucose, TGF-β_1_ and downstream CTGF were excessively secreted as 323.8 ± 14.2% and 286.8 ± 13.9%, respectively. Nevertheless, swinhoeic acid at 10 or 20 μM reversed the elevated secretion of these cytokines, which reached 253.7 ± 11.4% or 191.8 ± 16.3% for TGF-β_1_ and 225.8 ± 12.2% or 190.5 ± 14.0% for CTGF (Figure 3(D,E)).

### Swinhoeic acid activates Nrf2 in mesangial cells in the Keap1-dependent manner

**Figure 4.**
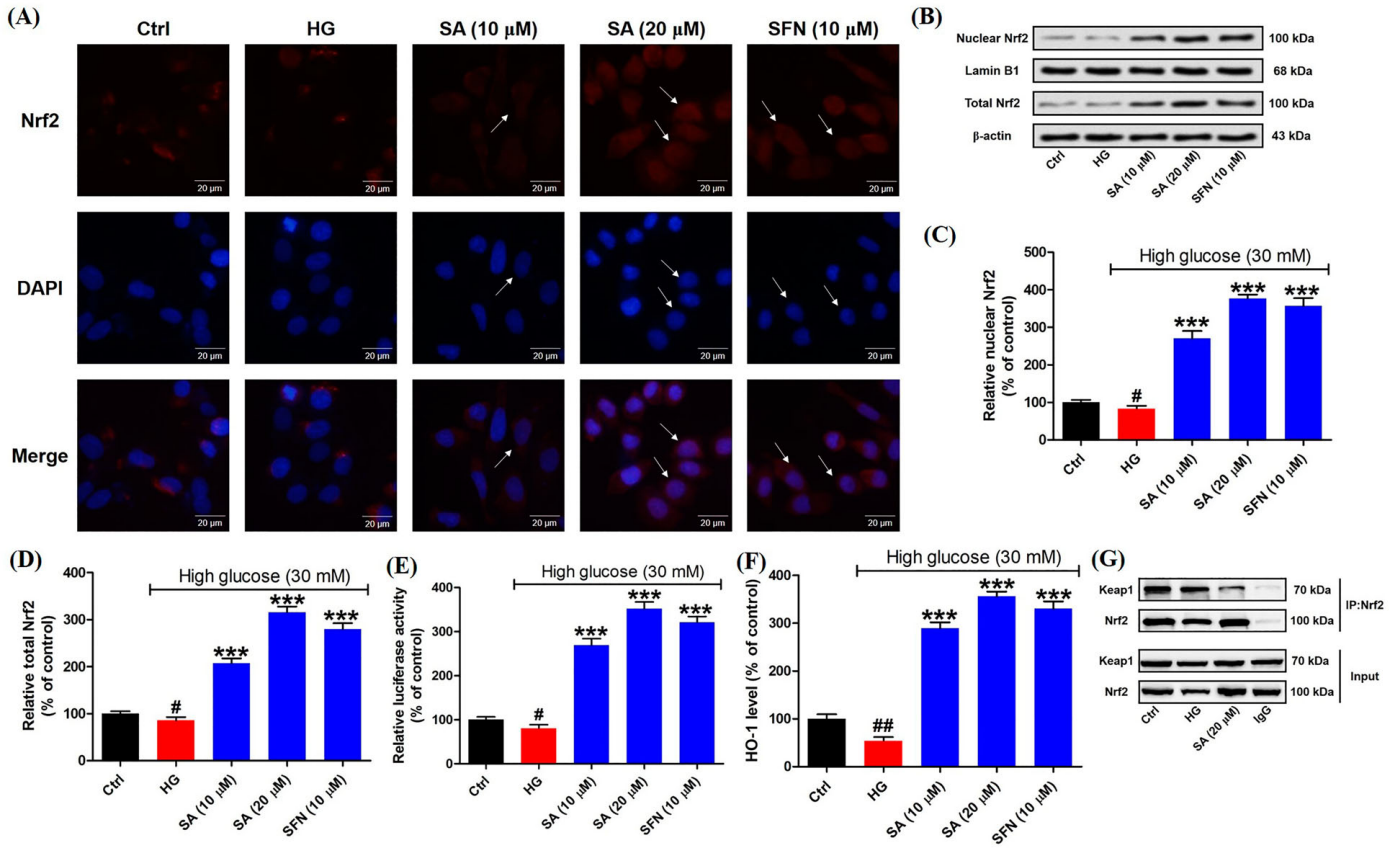
Effects of swinhoeic acid on the activation of Nrf2 in mesangial cells under high glucose. (A) Immunofluorescence staining, as the arrows indicated, the red fluorescence represented the intracellular Nrf2, blue fluorescence represented nuclei and the merged purple fluorescence represented the nuclear Nrf2. (B) Western blot analysis for nuclear and total Nrf2. (C)–(D) Densitometric analysis for nuclear and total Nrf2. (E) Dual luciferase assay for the capacity of Nrf2 binding to ARE. (F) HO-1 level. (G) Co-immunoprecipitation assay for Nrf2. *n* = 3, ^#^*P* < 0.05 and ^##^*P* < 0.01 *vs* control group, ***P < 0.001 *vs* high glucose group.

To explore the activation of Nrf2 in mesangial cells, firstly immunofluorescence staining was performed. The results indicated in the presence of swinhoeic acid, Nrf2 was enhanced in mesangical cells under high glucose. Particularly, swinhoeic acid at 20 μM has promoted the translocation of Nrf2 into nucleus from cytosol as the arrows indicated in the images, which is similar to the classical Nrf2 activator sulforaphane (Figure 4(A)). Then western blot analysis together with densitometric analysis for nuclear and total Nrf2 also revealed swinhoeic acid at 10 and 20 μM could stabilize free Nrf2 against its degradation and enhance its nuclear translocation (Figure 4(B–D)). At the same time, dual luciferase reporter assay disclosed swinhoeic acid could improve the transcriptional regulation of Nrf2 through the elevated capacity binding to ARE (Figure 4(E)). In addition, as the downstream ARE, the level of HO-1 was significantly up-regulated in the presence of swinhoeic acid or sulforaphane (Figure 4(F)). To elucidate the mode of Nrf2 activation, co-immunoprecipitation assay was implemented. As a result, it was observed that following the addition of swinhoeic acid, the level of Keap1 was reduced when Nrf2 was precipitated using itself primary antibody (Figure 4(G)), which unraveled swinhoeic acid could disrupt the Keap1-Nrf2 interaction to activate Nrf2.

### Activation of Nrf2 is involved in the effects of swinhoeic acid on mesangial cells

**Figure 5.**
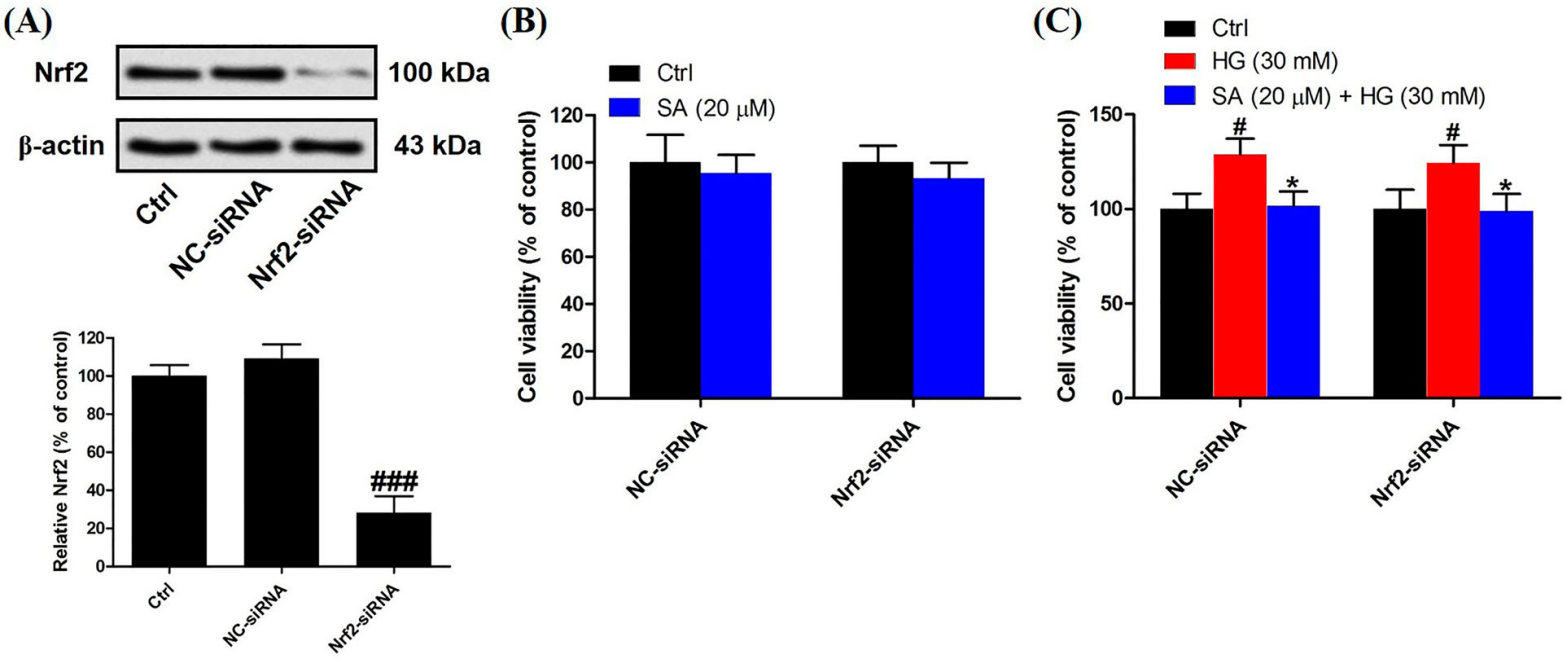
Nrf2 knockdown and effects on the survival of mesangial cells. (A) Western blot analysis and densitometric analysis for Nrf2 knockdown. (B) CCK-8 assay for the viability of mesangial cells transfected with NC-siRNA or Nrf2-siRNA cultured in normal medium. (C) CCK-8 assay for the viability of mesangial cells transfected with NC-siRNA or Nrf2-siRNA under high glucose. *n* = 3, ^#^*P* < 0.05, ^##^*P* < 0.01 and ^###^*P* < 0.001 *vs* control group, *P < 0.05 *vs* high glucose group.

**Figure 6.**
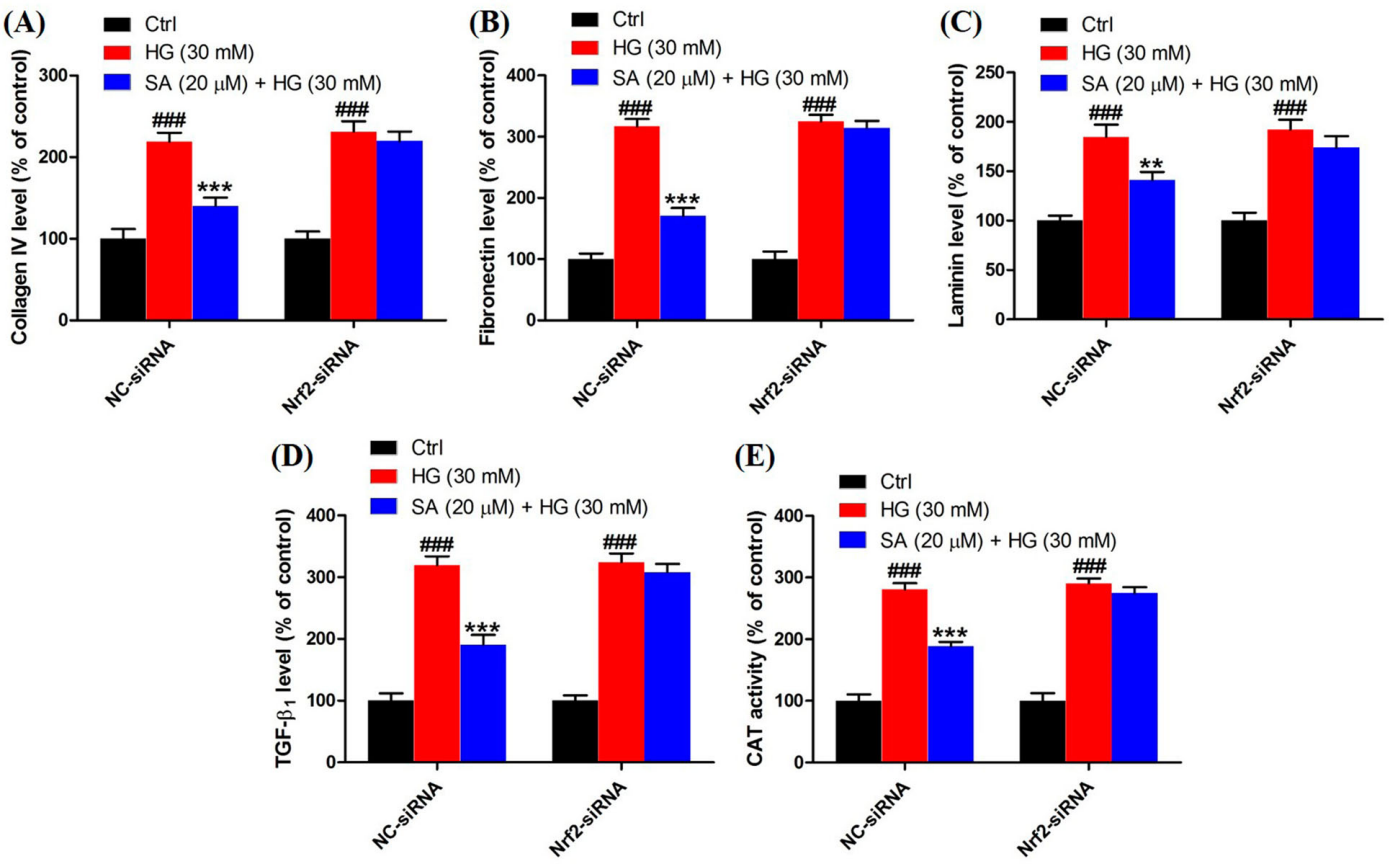
Role of Nrf2 activation in the effects of swinhoeic acid against accumulation of ECM. (A)–(C) Synthesis of collagen IV, fibronectin and laminin in mesangial cells transfected with NC-siRNA or Nrf2-siRNA under high glucose. (D)–(E) Secretion of TGF-β_1_ and CTGF in mesangial cells transfected with NC-siRNA or Nrf2-siRNA under high glucose. *n* = 3, ^###^*P* < 0.001 *vs* control group, **P < 0.01 and ***P < 0.001 *vs* high glucose group.

**Figure 7.**
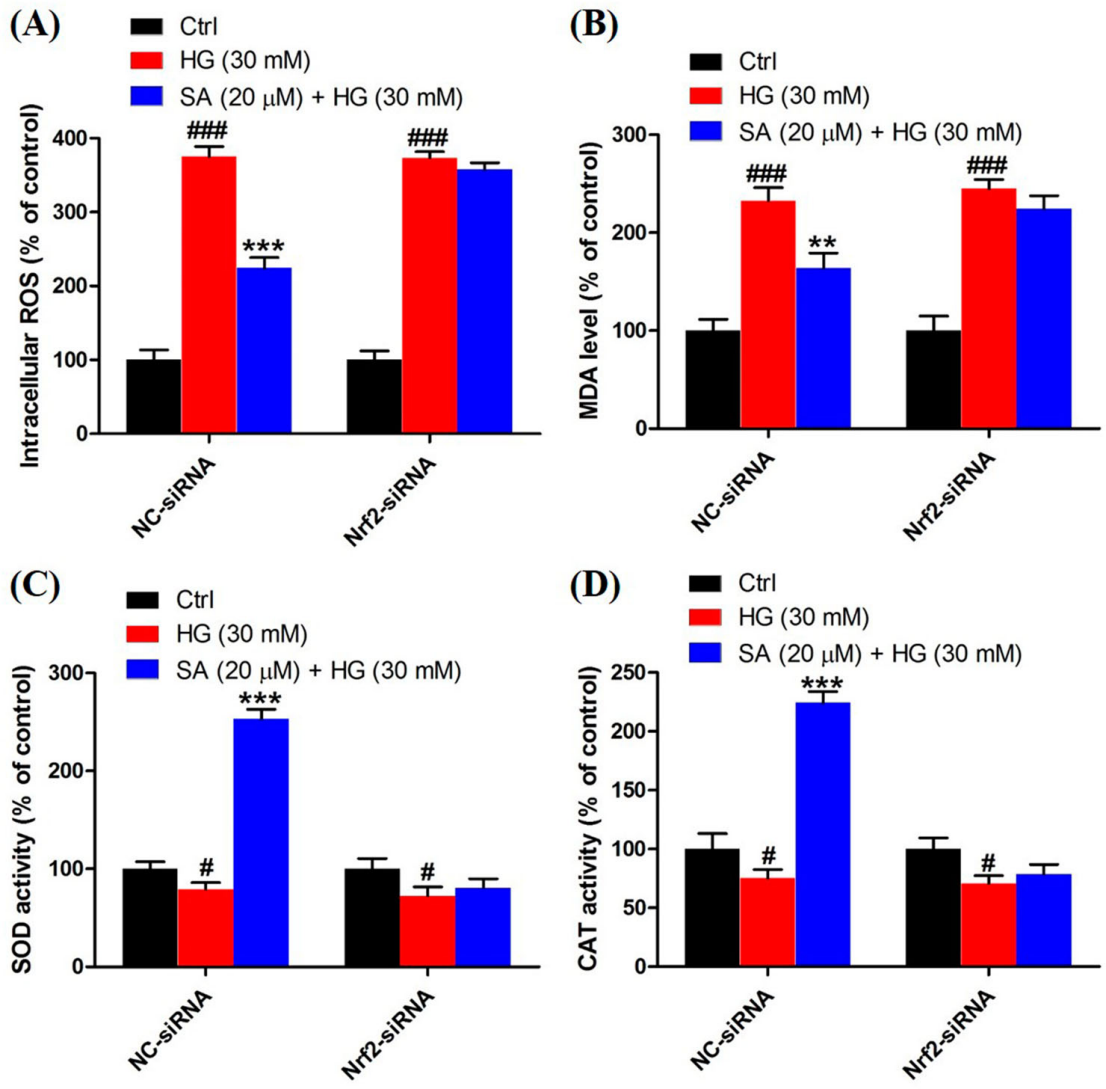
Role of Nrf2 activation in the effects of swinhoeic acid against oxidative stress. (A) Intracellular ROS level in mesangial cells transfected with NC-siRNA or Nrf2-siRNA under high glucose. (B) MDA level in mesangial cells transfected with NC-siRNA or Nrf2-siRNA under high glucose. (C)–(D) SOD and CAT activity in mesangial cells transfected with NC-siRNA or Nrf2-siRNA under high glucose. *n* = 3, ^#^*P* < 0.05 and ^###^*P* < 0.001 *vs* control group, **P < 0.01 and ***P < 0.001 *vs* high glucose group.

To validate the role of Nrf2 activation in the effects of swinhoeic acid, Nrf2 knockdown was employed using the siRNA interference. Western blot analysis together with densitometric analysis has indicated the transfection was attained successfully (Figure 5A). Meanwhile, it was found that transfection with Nrf2-siRNA didn’t affect the effects of swinhoeic acid on the viability of mesangial cells treated with or without high glucose (Figure 5(B,C)), which inspired us to further elucidate the role of Nrf2 activated by swinhoeic acid in oxidative stress and accumulation of ECM. ELISA assay has revealed swinhoeic acid couldn’t ameliorate the excessive synthesis of ECM proteins encompassing collagen IV, fibronection and laminin in mesangial cells following Nrf2 knockdown, whereas in mesangials cells transfected with NC-siRNA the effects of swinhoeic acid were also observed (Figure 6(A–C)). At the same time, the excessive secretion of TGF-β_1_ and CTGF induced by high glucose was not remarkably affected in the presence of swinhoeic acid in mesangial cells transfected with Nrf2-siRNA, while the effects of swinhoeic acid were also observed in the mesangial cells transfected with NC-siRNA (Figure 6(D–E)). In addition, further exploration showed swinhoeic acid couldn’t block the overproduction of intracellular ROS and MDA in mesangial cells transfected with Nrf2-siRNA, though its effects on mesangial cells transfected with NC-siRNA were not affected (Figure 7(A,B)). And the reduced activity of some antioxidant enzymes including SOD and CAT caused by high glucose in the mesangial cells was not elevated significantly by swinhoeic acid when Nrf2 was knocked down, but the activity in the cells treated with NC-siRNA was increased by swinhoeic acid (Figure 7(C,D)). These findings gave the evidence to demonstrate that activation of Nrf2 is associated with the inhibitory effects of swinhoeic acid against oxidative stress and accumulation of ECM.

## Discussion

As one of the most common complications of diabetes mellitus, DN is an important factor resulting in ESRD [1]. At an early stage of DN, high glucose is the important pathogenic factor of mesangial cells, which causes self-limited proliferation and following hypertrophy [19]. Herein, to find novel therapy for DN, we have found swinhoeic acid, a triterpenoid identified from *Potentilla fragarioides*, showed inhibitory effects on the proliferation of mesangial cells under high glucose.

In the pathogenesis of DN, oxidative stress resulting from the abnormal metabolism of high glucose plays a pivotal role and becomes the key link of signaling pathways associated with development and progression of DN [20]. In addition to cell injury, excessive ROS can promote the secretion of TGF-β_1_ and accumulation of ECM [21]. Superoxide is one kind of ROS and primarily yielded from one electron reduction of molecular oxygen [22]. Usually, it can be converted to hydrogen peroxide by SOD, and the latter can be degraded as water under the catalysis of CAT [23]. In the current investigation, we have found swinhoeic acid suppressed the intracellular ROS and the lipid peroxidation product, MDA, as well as activated SOD and CAT in mesangial cells under high glucose.

Fibrosis is the key character in the progression of DN, which is resulted from the accumulation of ECM [24]. As the key transforming growth factor, TGF-β_1_ enhances the synthesis and cross-linking of ECM [5]. CTGF is the downstream cytokine of TGF-β_1_, which is also involved in the secretion of ECM [25]. Under high glucose, mesangial cells will excessively synthesize ECM proteins and secrete TGF-β_1_ and CTGF [26]. Herein, we have observed swinhoeic acid inhibited excessive synthesis of ECM proteins as well as secretion of TGF-β_1_ and CTGF.

Nrf2 is a transcription factor regulating the expression of genes encoding antioxidant enzymes, and activation of Nrf2 will mitigate oxidative stress and fibrosis in diabetic kidney [27]. From the structure of Keap1-Nrf2 complex it has been revealed two Keap1 monomers will form a homodimer, and the latter will retain the DLG and ETGE motifs of Nrf2 using their Kelch domains, respectively [28]. Since the affinity of DLG to Kelch domain is lower than ETGE, the Keap1 homodimer will form a ‘hinge and latch’ model with Nrf2 [29]. As the oxidative stress sensor, thiol groups in cysteine residues of Keap1 can be modified by oxidants or electrophiles, which will result in conformation changes of Keap1 followed by dissociation of DLG from Keap1 [30]. Accordingly, it is hindered that Keap1 facilitates ubiquitination and degradation of Nrf2 as an adaptor. In our investigation, it was observed that swinhoeic acid activated Nrf2 via interacting with Keap1, which is closely associated with its effects on mesangial cells against oxidative stress and accumulation of ECM. From the structure of swinhoeic acid, it can be found there is a moiety of conjugated olefin adjacent to an electron-withdrawing carboxyl group as the Michael acceptor. Michael acceptors are representative electrophiles, since they belong to the soft Lewis acids. And they can react with the key thiol groups (soft base) in cysteine residues of Keap1 through Michael addition reaction, which gives rise to the activation of Nrf2 [31]. Therefore, it can be proposed swinhoeic acid is a Keap1-dependent Nrf2 activator. In addition, there are some reductive moieties including carbonyl, hydroxyl and alkenyl in swinhoeic acid, which show the potential to react with oxidants such as ROS to scavenge them. And this may be another mechanism for the antioxidant activity of swinhoeic acid, which should be further explored in future investigations.

## Conclusion

**Figure 8.**
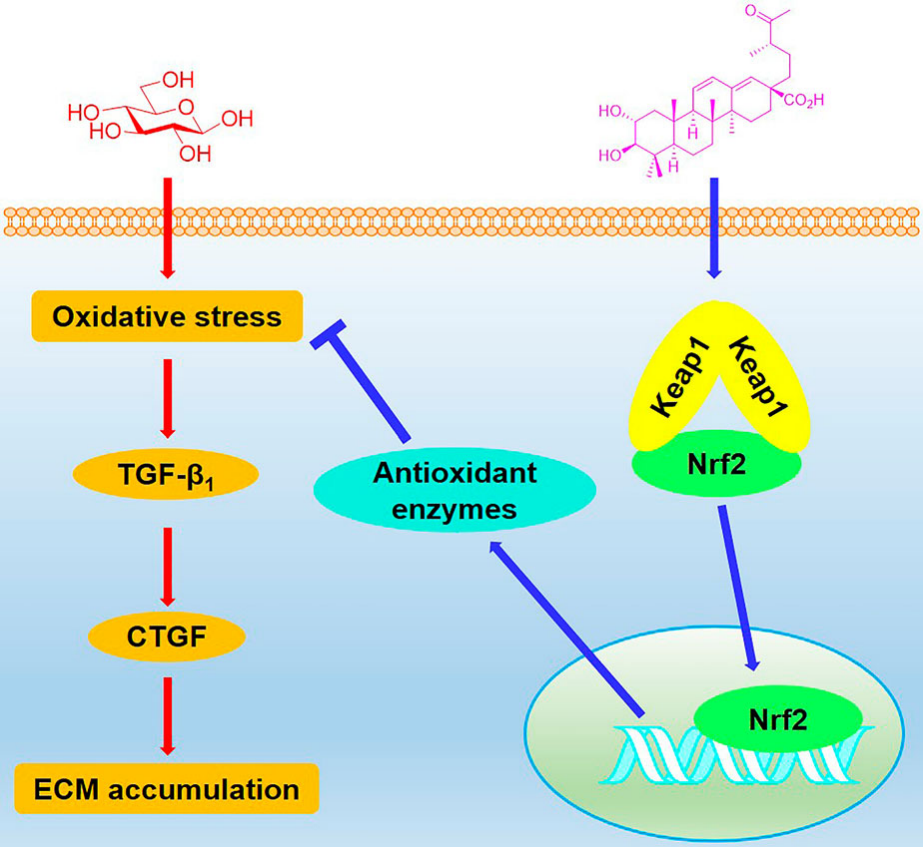
Schematic illustration for the effects of swinhoeic acid on mesangial cells under high glucose.

In conclusion, we have explored the effects of swinhoeic acid on mesangial cells under high glucose. In addition to the inhibition of self-limited proliferation, swinhoeic acid attenuates oxidative stress and accumulation of ECM resulting from high glucose, which is closely associated with the Keap1-dependent activation of Nrf2 (Figure 8). These findings provide evidence for the discovery of Nrf2 activators and therapy for DN.

## Data Availability

The data in the current study are available from the corresponding author upon reasonable request.
